# Three-dimensional printed models as an effective tool for the management of complex congenital heart disease

**DOI:** 10.3389/fbioe.2024.1369514

**Published:** 2024-08-02

**Authors:** Katia Capellini, Lamia Ait-Ali, Vitali Pak, Massimiliano Cantinotti, Michele Murzi, Emanuele Vignali, Benigno Marco Fanni, Alberto Clemente, Simona Celi, Emanuele Gasparotti

**Affiliations:** ^1^ BioCardioLab, Bioengineering Unit, Fondazione Toscana Gabriele Monasterio, Massa, Italy; ^2^ Institute of Clinical Physiology, CNR, Massa, Italy; ^3^ Department of Pediatric Cardiac Surgery, Fondazione Toscana Gabriele Monasterio, Massa, Italy; ^4^ Department of Pediatric Cardiology, Fondazione Toscana Gabriele Monasterio, Massa, Italy; ^5^ Department of Adult Cardiac Surgery, Fondazione Toscana Gabriele Monasterio, Massa, Italy; ^6^ Department of Clinical Imaging, Fondazione Toscana Gabriele Monasterio, Pisa, Italy

**Keywords:** 3D printing, 3D models, congenital heart disease, pediatric surgery, pre-planning

## Abstract

**Introduction:**

Three-dimensional printed models are widely used in the medical field for surgical and interventional planning. In the context of complex cardiovascular defects such as pediatric congenital heart diseases (CHDs), the adoption of 3D printed models could be an effective tool to improve decision-making. In this paper, an investigation was conducted into the characteristics of 3D printed models and their added value in understanding and managing complex pediatric congenital heart disease, also considering the associated cost.

**Methods:**

Volumetric MRI and CT images of subjects with complex CHDs were retrospectively segmented, and the associated 3D models were reconstructed. Different 3D printing technologies and materials were evaluated to obtain the 3D printed models of cardiac structures. An evaluation of time and costs associated with the 3D printing procedure was also provided. A two-level 3D printed model assessment was carried out to investigate the most suitable 3D printing technology for the management of complex CHDs and the effectiveness of 3D printed models in the pre-surgical planning and surgical strategies’ simulations.

**Results:**

Among the different techniques, selective laser sintering resulted to be the most suitable due to its reduced time and cost and for the positive clinical feedback (procedure simulation, surface finish, and reproduction of details).

**Conclusion:**

The adoption of 3D printed models contributes as an effective tool in the management of complex CHDs, enabling planning and simulations of surgical procedures in a safer way.

## 1 Introduction

Three-dimensional printing technology in medicine has developed very rapidly in recent years due to the several approaches where it can be applied and the provided advantages (Bozkurt and Karayel, 2021; Javaid et al., 2022). In the cardiovascular field, 3D printed models are used for several purposes such as medical students’ education and training, patients’ communications, planning of surgical and percutaneous interventions, and applications in mock circulatory systems to investigate fluid dynamics *in vitro* (Medero et al., 2017; Vukicevic et al., 2017; Buonamici et al., 2022; Santoro et al., 2022; Vignali et al., 2022; Fanni et al., 2023; Masoumkhani et al., 2023; Stomaci et al., 2023).

This great development is strictly correlated with the advance in technology of volumetric medical imaging, and the resolution and signal-to-noise ratio are indeed crucial aspects to obtain an accurate 3D printed model. A certain demarcation between the structures of interest and the other image regions is one of the main starting points in the process of 3D model reconstruction (Fan et al., 2019). Although 3D echocardiography concerns mostly 3D models of cardiac valves and small defects due to a limited field of view, computed tomography (CT) and magnetic resonance imaging (MRI) are the most diffused acquisition techniques suitable for 3D model generation of several cardiovascular pathological anatomies. CT images are characterized by high spatial resolution and contrast; however, their adoption is not recommended for pediatric subjects, given the necessity to inject an iodinated agent to enhance contrast and the ionizing nature of the radiations involved. On the other hand, MRI does not involve ionizing radiation and presents a good tissue to blood contrast but is affected by a poorer spatial resolution and more artifacts compared to CT (Celi et al., 2021).

Starting from the elaboration of these aforementioned medical images, 3D printed models can be realized with several kinds of 3D printing technologies and materials, resulting in different characteristics in terms of quality, color, opacity, deformability, time, and costs (Gharleghi et al., 2021).

The most commonly used 3D printing technologies include fused deposition modeling (FDM), stereolithography (SLA), and selective laser sintering (SLS).

Regarding FDM technology, the material is deposited in layers to form a 3D object by adopting a thermoplastic filament melted from a heated nozzle (Awasthi and Banerjee, 2021). FDM is adopted in several works evaluating the inclusion of 3D printed models in the surgical planning of cardiac defects (Valverde et al., 2017; Capellini et al., 2020).

The SLA technique consists in a UV laser light that induces a polymerization process with a tank filled with photo-polymeric resin. SLA technology allows for only one material printing at a time, and external and internal supports are required to prevent model collapse. Rigid or soft resin materials can be used, allowing the adoption of SLA in cases of training (Mafeld et al., 2017) and pre-planning for the percutaneous procedure (Capellini et al., 2020; Kaufmann et al., 2023).

SLS involves the powder bed fusion technology, which utilizes the laser energy to heat and melt powder particles (Lekurwale et al., 2022). SLS technology allows for only one material printing at a time, and it does not require the adoption of external and internal supports during the fabrication process (Kappanayil et al., 2017). It is possible to adopt opaque rigid or soft materials.

Congenital heart disease (CHD) is one of the most widespread congenital pathology in infants (Wu et al., 2020). In particular, complex CHDs consist in the concomitant presence of more morphological defects (Festa et al., 2023), and they are characterized by uncommon anatomic relations; often, they require complex surgical approaches. The role of 3D models in the CHD understanding and surgery is well-established in the literature, together with their utility in teaching, training, and communication (Capellini et al., 2020; Awori et al., 2021; Karsenty et al., 2021). Although there are some studies in the literature that highlight the benefits of 3D virtual models in the field of CHDs (Ong et al., 2018; Awori et al., 2023), the adoption of 3D printed models is also able to provide a tactile response and more realistic understanding of depth and anatomic relationships with surgeons (Costello et al., 2015; Celi et al., 2021; Illi et al., 2022).

However, clinicians’ feedback arising from both the effectiveness in the decision-making procedure for pediatric complex CHDs and the different kinds of 3D printing technologies and materials needs further investigation. This study aims to assess the additional value of 3D printed models in the pre-planning of complex CHDs as well, with respect to different 3D printing techniques and materials.

## 2 Materials and methods

### 2.1 Image data

**FIGURE 1:**
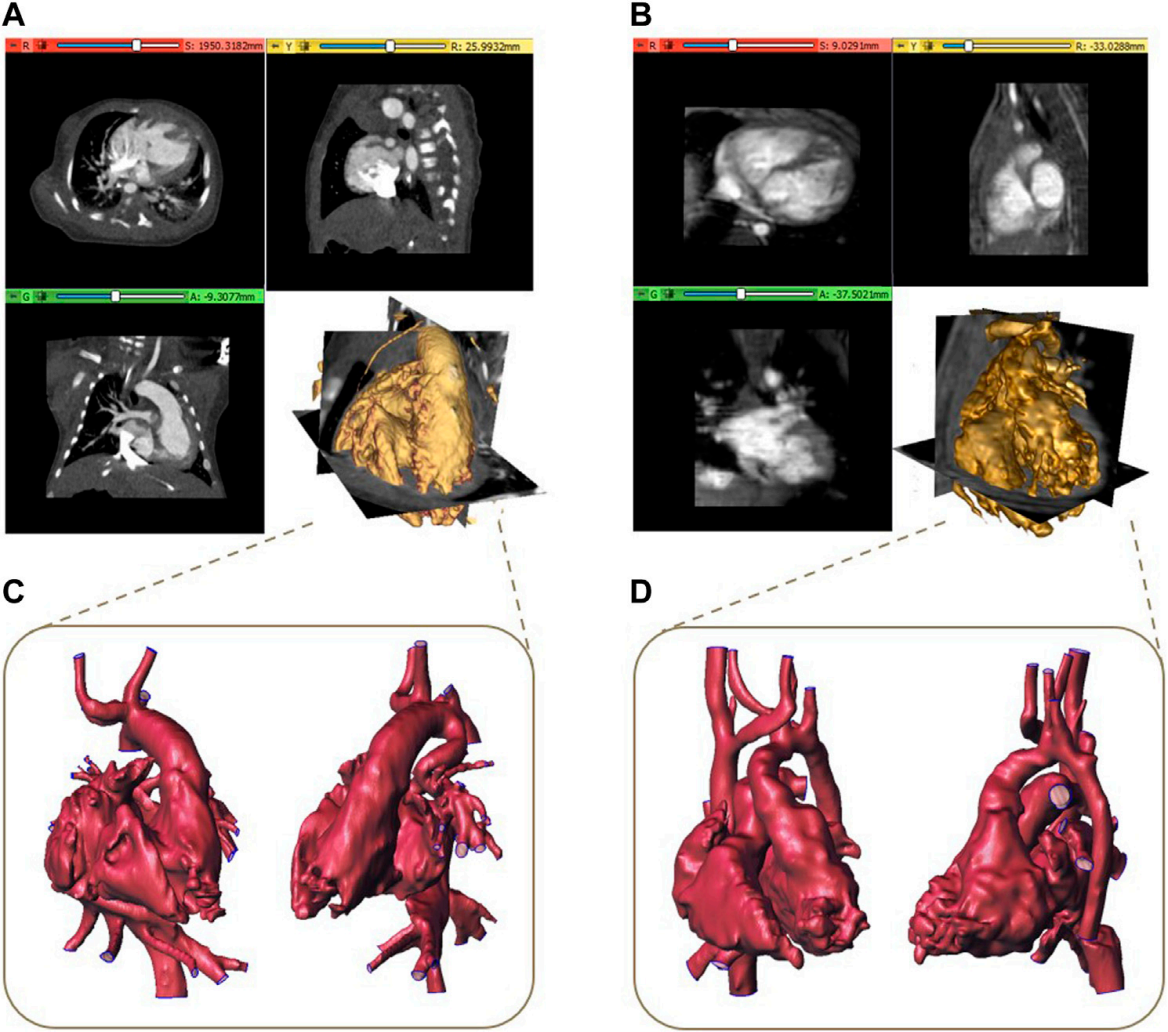
Examples of CT **(A)** and MR dataset **(B)** with the associated volume rendering visualization and the corresponding reconstructed 3D models **(C, D)**.

Ten pre-surgical image datasets of patients (five male subjects and five female subjects with an average age of 4 years) presenting complex CHDs scheduled for the surgical procedure were retrospectively analyzed in this study. In particular, two volumetric CT and eight MRI datasets acquired with 320-detector scanner (Toshiba Aquilion One, Toshiba, Japan) and 3 Tesla scanners (Ingenia, Philips Medical Systems, Netherlands), respectively, were considered. Examples of CT and MR datasets with the associated volume rendering representations are shown in Figures 1A, B, respectively.

### 2.2 Image processing

After an operation of image cropping to reduce the dataset volume to the region of interest, a segmentation process is required to obtain the 3D model of the anatomical structures. All the segmentation procedures described below were implemented by using 3D Slicer, a free and open-source software application (www.slicer.org) for the analysis and segmentation of biomedical images (Kikinis et al., 2013). A threshold algorithm was initially applied for the segmentation of both CT and MRI datasets, given the presence of contrast between blood in the vessels and heart chambers and non-vascular tissues. Secondary, a semi-automatic technique based on the region growing algorithm was applied together with a final phase of manual editing, often consisting in a slice by slice segmentation, due to the difficulty for automatic methods to accurately identify regions with complex defects or artifacts. This procedure was carried out by highly specialized biomedical engineers in the cardiovascular field with specific attention to cardiac structures with more than 5 years of experience in complex CHD segmentation with the support of clinicians with expertise in cardiac imaging. Starting from the obtained segmentation binary mask, a preliminary 3D model was reconstructed and then refined by performing detection and correction of any mesh holes, a removal of non-manifold edges and islands. Examples of 3D final models are reported in Figures 1C, D. To obtain the final 3D model suitable for printing, for all the models, the surfaces were thickened of 0.8 mm outward from the mesh to maintain the actual dimensions of the reconstructed blood cavities.

### 2.3 Three-dimensional printing technologies

Three different types of 3D printing technology were tested in this study: FDM, SLA, and SLS.

First, a subset of three 3D models was realized with all aforementioned 3D printing technologies to perform an accurate comparison and evaluation of the most suitable technique for the complex CHDs. Finally, the remaining 3D models were realized with the chosen technique. Flexible materials with a comparable shore, approximately 80 A, were chosen, given its availability for each considered 3D printing technology and its applications for the realization of anatomical models (Gómez-Ciriza et al., 2021; Lau et al., 2021; Lau and Sun, 2022; Kaufmann et al., 2023). Regarding the FDM technology, a G-code file was generated by using slicing SSI software (3ntr, Oleggio, Italy), allowing the definition of the model position and orientation on the printing plate and the selection of supports necessary for the printing. An A4v4 printer (3ntr, Oleggio, Italy) was adopted (printing volume: 30.0×17.1×20.0 cm^3^). The model was printed in a thermoplastic polyurethane printing filament (TPU Elasto85) with shore 85 A. With regards to the printing parameters, the thickness of the layer was chosen according to the selected material. An adaptable layer ranging from 0.15 mm to 0.25 mm was adopted together with an infill of 100%, given the thickness of the model. A polyvinyl alcohol (PVA) material (SSU04) was used as support, given its water-soluble properties. The post-processing steps consisted in the manual removal of the external support and then by using a tank filled of warm water with ultrasound enabled to dissolve the internal support. The FDM technology needs approximately 310×90 cm to house the printer, the ultrasound washer, and a workbench for the manual operations. The expanses of this technique include the equipment cost (evaluated equal 0.8 €/h), the material cost (Elasto85 = 104 €/kg, SSU04 = 121 €/kg), and the post-processing equipment cost (estimated 0.1 €/h).

Three-dimensional printing with SLA technology was allowed by using Preform software (Formlabs, Somerville, United States). The position and the orientation of the model were defined to allow the easier removal of supports and guarantee the highest quality of the printed details. The SLA models were fabricated through Form 3BL printer (Formlabs, Somerville, United States), characterized by a maximum printing volume of 33.5×20.0×30.0 cm^3^. The model was printed in a deformable resin (Flexible 80 A) with a shore value of 80 A. Each model was realized with a layer thickness of 0.1 mm, compatible with the selected material. Post-processing of SLA consisted of cleaning the model from uncured resin by a washing procedure using isopropyl alcohol, then curing in an ultraviolet and warm environment, and finally the removal of the outer and inner supports by using proper scissors.

The SLA requires approximately 450×90 cm of space to accommodate the printer machine, the washer, the curer, and a workbench for the post-processing procedures. The equipment cost of SLA was evaluated to be equal to 1.25 €/h for the printing process and the material cost of Flexible 80 A was 242.8 €/kg, whereas the equipment cost for the post-processing was estimated to be equal to 0.42 €/h.

The SLS technology was defined by using Sinterit Studio Advanced (Sinterit, Krakow, Poland), where the position and the orientation of each model were set to guarantee the highest quality of the model and reduce the printing time. The SLS CHD models were realized through a Lisa PRO printer (Sinterit, Krakow, Poland) with a printing volume of 11×15× 25 cm^3^. The material used was Flexa Bright, with 79 A of shore. Each model was realized with a layer thickness of 0.125 mm. SLS post-processing consisted of cleaning the machine using the unsintered powder, removing the powder deposited on model surfaces during printing by vacuum suction, air sanding, and finally cleaning with soap and water. The SLS needs approximately 450×90 cm to host the printer, the powder sieve, the sandblaster, the vacuum cleaner, and a workbench for the manual operations. The equipment cost of SLS was evaluated to be equal to 1.7 €/h, whereas the material cost of Flexa Bright was 250 €/kg and the post-processing equipment cost was estimated to be 0.36 €/h.

Supplementary Table S1 summarizes the main features of 3D printing technologies adopted in this work. Regarding the 3D printing equipment, the hourly costs were calculated considering a lifespan of 5 years (15,000 h). In addition to the abovementioned costs, the cost of the printer operator associated with manual activities performed before and after the printing process was also considered. The operator involved in this study had more than 1 year of experience in the 3D printing technologies applied for cardiovascular anatomies, and the associated hourly labor cost was 20 €/h (it is important to report that this cost depends on each country and legislation). The total print time, encompassing setup time (for G-code generation and printer preparation), printing time, and post-processing time were assessed.

### 2.4 Evaluation of 3D printed models’ effectiveness

A two-level 3D printing effectiveness evaluation was carried out. The effectiveness was evaluated by a clinical team that did not take part in the pre-planning and surgical phases of the selected cases. The team was composed by a total of six physicians, of which three were cardiologists and three were pediatric surgeons. The first level of evaluation assessed which adopted 3D printing technologies and materials were the most suitable for the management of complex CHDs. A subset of 3D models, including the most representative cardiac defects, such as transposition of the great arteries, crisscross heart, and ventricular and atrial septal defects, was chosen for the evaluation and printed with the considered technologies. In particular, the clinical team assessed three different parameters:

• Surface—surface finishing.

• Details—the capability to replicate anatomical details with high accuracy.

• Behavior—the material feedback for the simulation of the surgical procedure, considering the level of deformability and the behavior of the model to be cut with the scalpel.

Each member of the clinical team was asked to assign a score from 1 to 5 for these parameters for each printed model. In this way, each technology was evaluated on the basis of 54 scores (three parameters × three models × six clinicians), with 18 scores for each parameter, arising from the clinicians rate to each printed model.

The 3D printing technology characterized by the best scores in the first assessment was selected to realize all 10 cases included in the second level of evaluation. This approach allowed cost and time savings by avoiding the adoption of 3D printing technologies that received lower ratings from clinicians. For each model, the pre-planning of the surgical procedure was carried out by the clinical team. In particular, in this phase, the surgical strategy was defined considering for each case, at first, only the available clinical images (a); then, a strategy reassessment was performed including the 3D printed model as well (b). In this way, the impact of 3D printing on the management of complex CHDs was estimated by investigating whether and how the additional information provided by the 3D printed model modify the course of surgical planning and eventually the surgical decision. In particular, the clinical team evaluated the possibility to better understand the pathology as well by exploring internal heart chambers from different views, the time of pre-planning, and the potential improvement in the communication between clinicians. The evaluation was performed on all CHD models. The clinical team was required to assign a rating (“don’t know,” “worsen,” “irrelevant,” “quite relevant,” “relevant,” and “very relevant”) in a specific survey.

## 3 Results

### 3.1 Image processing

Regarding the segmentation process, manual editing was necessary in most of the MRI datasets, especially in the cases of newborns due to the complexity of structures and presence of motion artifacts. However, for the CT datasets, which required further segmentation phase after the threshold algorithm application, semi-automatic methods limited to restricted volume regions were preferred due to the higher spatial resolution and size of datasets. The 3D models were successfully reconstructed for all selected image datasets, and the segmentation times requested for the generation of 3D models are reported in Table 1. The time values varied depending on the complexity of CHDs, the image contrast and resolution, and the type of adopted segmentation algorithm, ranging between 4.5 and 10.4 h. Table 1 also reports the printing volume associated with the segmented 3D models, and it is possible to observe that the dimensions of the considered models allow the adoption of the considered printing technologies.

**TABLE 1:** Image processing and 3D printing characteristics. “ − ” means no printed model for the technique.

Case	Image modality	Segmentation time (h)	Printing volume (cm^3^)	Printing time (h)		
				FDM	SLA	SLS
1	MR	7.3	8.6 × 6.2 × 11.2	19.3	19.5	16.0
2	CT	9.2	6.5 × 5.8 × 9.7	10.7	15.0	10.3
3	MR	10.4	8.6 × 6.9 × 10.2	17.0	17.1	14.0
4	MR	4.5	9.8 × 6.7 × 12.5	−	−	19.1
5	MR	5.3	10.5 × 9.3 × 14.3	−	−	23.5
6	MR	8.1	10.6 × 7.0 × 13.0	−	−	21.2
7	MR	10.4	8.9 × 6.7 × 7.6	−	−	13.0
8	CT	8.5	8.4 × 6.1 × 8.5	−	−	14.5
9	MR	7.5	10.6 × 9.3 × 13.2	−	−	25.0
10	MR	7.3	14.4 × 10.5 × 9.6	−	−	20.0

### 3.2 Three-dimensional printing

**FIGURE 2:**
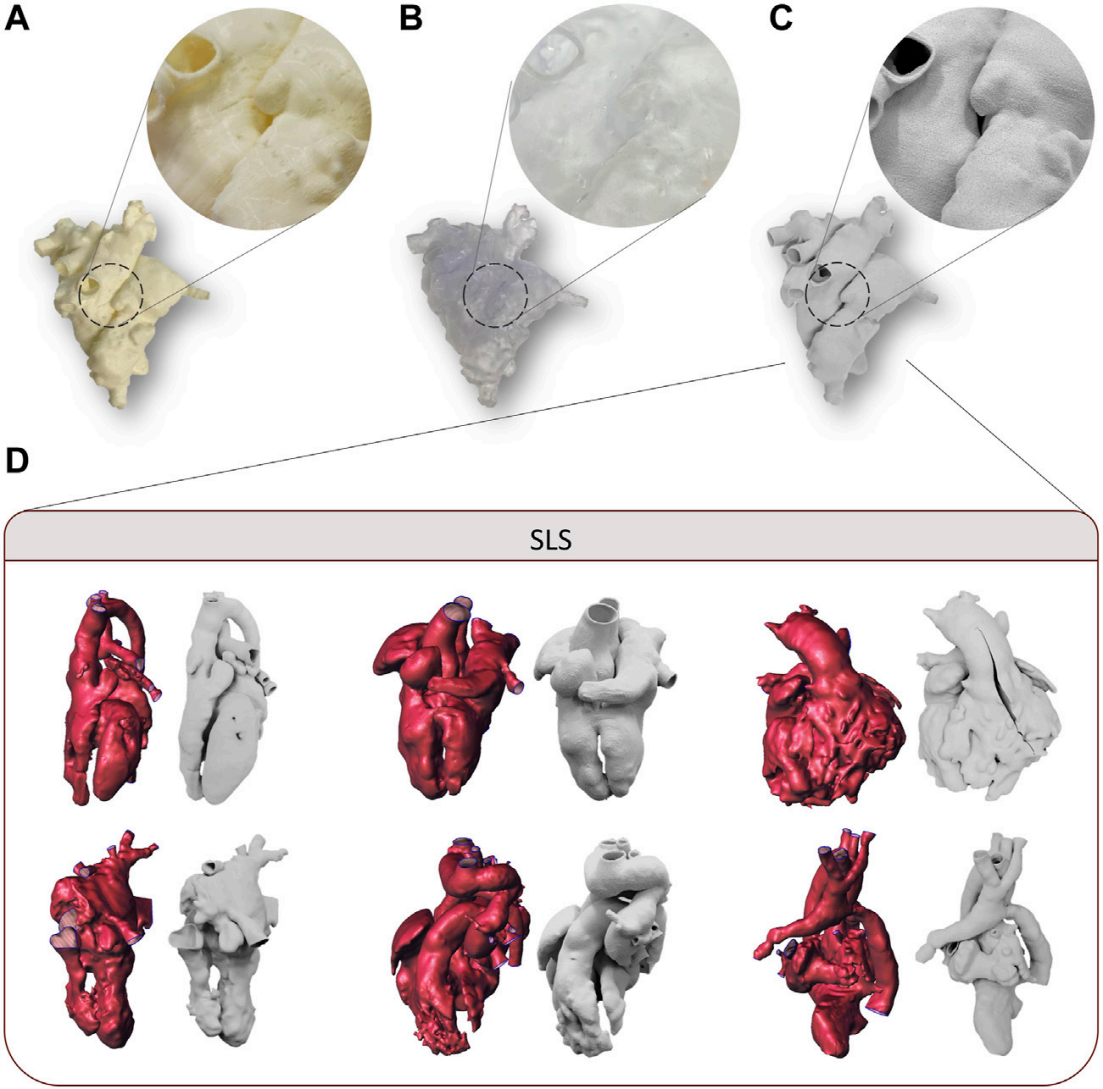
Examples of the 3D printed model of case 3 by using FDM **(A)**, SLA, **(B)** and SLS **(C)** for first level of evaluation, with detail magnification. Examples of 3D models and the corresponding 3D printed models of cases manufactured with SLS and used for second level of evaluation **(D)**.

FDM technology allowed the printing of all selected models without complications related to anatomical complexity, reproducing both the external and the internal structures without macroscopic defects (Figure 2A). The printing time to realize the models with FDM technology increased with the increasing printing volume of the model, showing a mean value of 15.7 h (Table 1). The post-processing time was approximately 6 h, considering a washing time of 5 h, and it is independent from the printing volume in the range of complex CHDs considered. The cost related to the FDM process is proportional to the printing volume with a mean value of 77 €.

SLA technology allowed the printing of all selected models, although some critical issues arose in the case of small complex structures characterizing the CHDs. In fact, for all the models analyzed, the main issues were found in the post-processing phase. In particular, the extraction of supports without damaging the model was made difficult by the need to use high-density supports for internal structures, the small size of the models, and the lack of holes of access usable for the scissors. In addition, given the presence of high-density support, support imprints on the surface of the models were observed (Figure 2B). The printing time of SLA technology increased with the increasing printing volume of the model, showing a mean value of 17 h (Table 1). The post-processing time was approximately 2 h, considering 30 min for model washing and curing, and the support manual remotion is dependent on the printing volume and the degree of complexity of the CHDs. The cost related to the SLA process is proportional to the printing volume, with a mean value of 81€.

The realization of all 3D models was feasible through the SLS printer. The relatively small dimensions of the printing plate, in fact, did not represent a limitation for the printing because of the small size of pediatric heart models. SLS was able to reproduce both the external and the internal structures of the 3D model without macroscopic defects, with accurate adhesion of the printer layers (Figure 2C). As observed for the other two technologies, SLS exhibited printing time proportional to the model volume, with a mean value of 13.4 h (Table 1). The post-processing time was independent from the model weight, and it was equal to 1 h. The mean cost related to SLS was equal to 56€. Some examples of 3D printed models by SLS are reported in Figure 2D.

### 3.3 Evaluation of 3D printed models’ effectiveness

**FIGURE 3:**
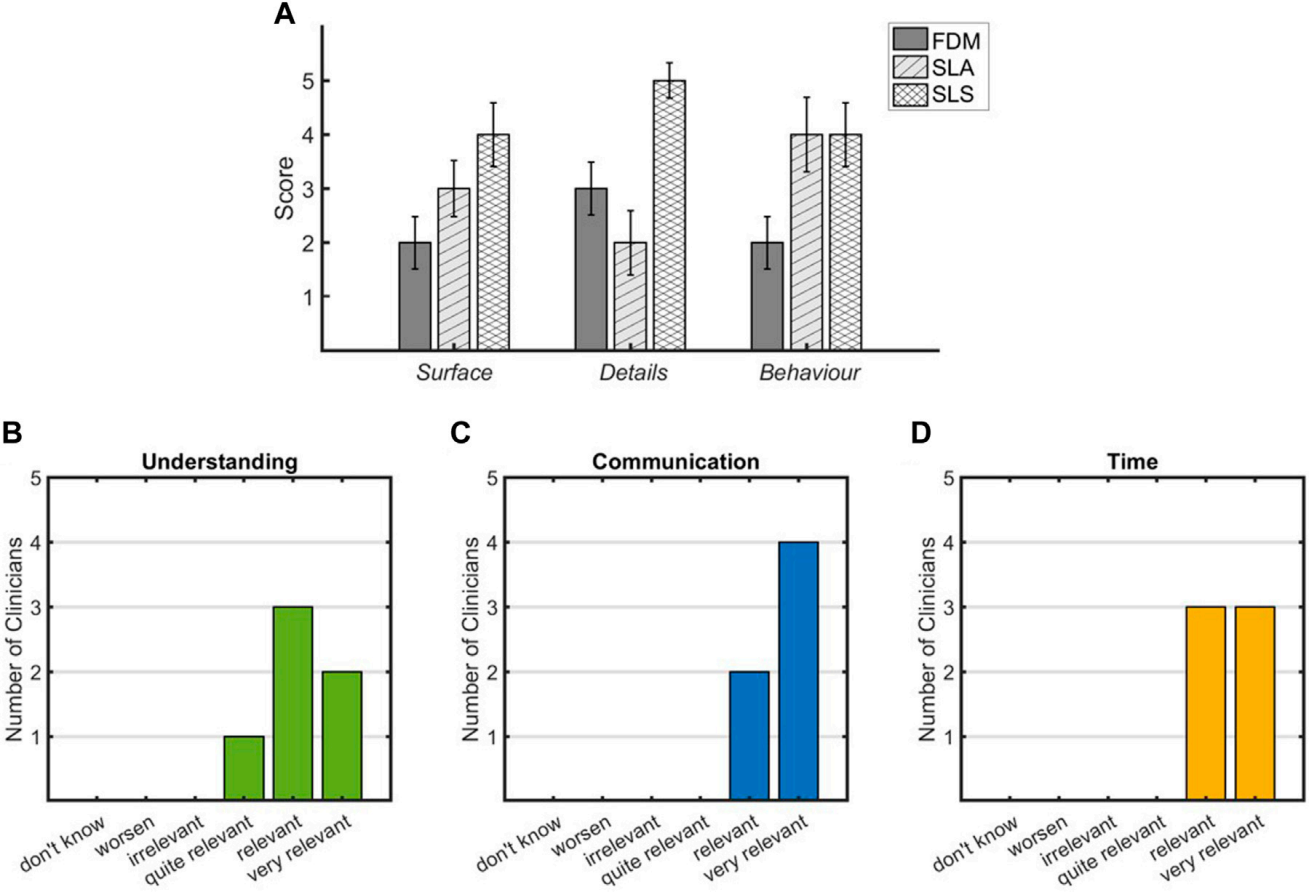
Reports of the clinical team evaluation for 3D printing technologies (first level of evaluation) **(A)** and for the impact of 3D printed models on pre-planning (second level of evaluation) **(B–D)**.

Regarding the comparison of the tested 3D printing technologies, the mean value of the scores assigned by the clinical team is shown in Figure 3A. The team assigned the highest value of *Surface* score to the SLS technique as it presented satisfactory layer adhesion and good surface finish. SLA achieved a *Surface* rating of 3 as the imprint of the removed supports was present on some regions of the external surface. The FDM reached the lower value as it presents a coarse surface with visible layers. In terms of the *Details* parameter, the SLS achieved the highest value as the anatomical details were reproduced with high quality on both the external and inner surfaces. The FDM model showed a lower quality of details on the internal surfaces, while the SLA model reached the lowest value of 2 as the inspection of the internal details was limited by the presence of the support and the transparency of the model. The last parameter was *Behavior*; the SLS and SLA scored the highest because the elasticity offered allowed surgical incision and inspection without damaging the model. In addition, the material compliance was more similar to cardiac tissue than the FDM material when handled by the surgeon. The lower score for FDM (equal to 2) was due to the stiffer behavior of the material, which made the cutting phase more difficult.

As concerns for the second-level assessment, the survey was completed by the clinicians for all cases. Figures 3B–D report the results in terms of rating assigned to each parameter. Regarding the understanding of individual complex CHDs, all experts agreed that SLS-printed models provide an increase in the comprehension of anatomical details and relations that characterize the pathology, as reported in Figure 3B, where five out of six clinicians assigned a rate of “relevant” and “very relevant.” All clinicians were agreed on the relevance of the SLS-printed model in the communication among physicians during the decision-making procedure (Figure 3C). The inclusion of the 3D printed model in the clinical discussion showed a relevant impact on the time needed for the decision-making procedure that resulted to be reduced (Figure 3D). The importance of performing strategies’ simulations on the 3D printed model was highlighted in eight cases, and for 4 out of 10 cases, the surgical strategy resulted different, compared to that performed based only on clinical images.

## 4 Discussion

In this work, an investigation of the main diffused 3D printing technologies and added value of 3D printed models in the management of complex CHDs was carried out. In the last few years, the adoption of 3D virtual and printed models in the cardiac field has significantly increased (Gasparotti et al., 2019; Gardin et al., 2020; Ma et al., 2021). In particular, their usage can be crucial in the understanding, pre-planning, and simulation of surgical procedures of pediatric complex CHDs.

In our study, 10 complex CHD anatomies were analyzed starting from the segmentation of medical images, up to 3D printing realization and clinical evaluation to assess the applicability of the 3D printed models in pre-planning. The clinical images play a central role in the process of 3D model realization, in particular MR and CT acquisitions are the most used in the cases of complex CHDs. In addition to the advantages and drawbacks associated with the above cited imaging modalities and already described in Section 1, the proper balance between spatial resolution and image contrast is a crucial aspect. A good resolution in terms of small voxel size allows the visualization and reconstruction of small structures details but involves a decrease in the signal-to-noise ratio with consequent difficulties associated with segmentation algorithm application (Otton et al., 2017).

In the context of a clinical application, the total print time is a parameter that characterizes the availability of the printed model, following a clinical request. All considered techniques result in compliance with the clinical practice, allowing the model to be available to the clinician within 24 h on average after the segmentation procedure (7.8 h). Among the techniques, the SLS resulted to be the fastest method, requiring 34% and 28% less time with respect to FDM and SLA. This is related to the smaller amount of material used for printing due to the absence of supports.

Moreover, the spaces required by the 3D printing techniques inside a clinical environment have to be taken into account. The considered 3D printing technologies require a comparable amount of space, and a dedicated room should be needed for any of them due to the characteristics of materials involved in setup, printing, and post-processing phases. In addition, the cost associated with 3D printing is an important aspect discussed in the literature (Lau et al., 2019; Yoo et al., 2021) since it represents one of the discriminating factors for the inclusion of this procedure in the clinical routine management of CHDs. Other technologies are widespread in the field of 3D printing such as PolyJet technology by Stratasys that permits the realization of 3D models varying the shore values of the printed structures. In particular, the Digital Anatomy printer is used to create cardiac structures with different stiffness. Nevertheless, these other technologies have an equipment acquisition cost of over 70 k€ (Chae et al., 2015; Chen et al., 2021), while in this work, we focused on 3D printer machines available in our center, characterized by a cost lower than 30 k€. The tested 3D printing technologies were selected, taking into account both the technical characteristics associated with the printing models and the associated cost-effectiveness that is fundamental in a clinical scenario. From the evaluation of the costs of the 3D printing technologies tested in our work, it appears that, even if the cost of producing a model is similar for the analyzed technologies and therefore compatible with clinical application, the SLS process is the cost-effective method with a cost-benefit ratio 28% and 31% lower than FDM and SLA, respectively. Although SLS has the highest cost of material per kilogram, this technology minimizes material waste, and the costs for post-processing are the lowest due to the absence of supports.

The accuracy in the delineating anatomical details is an explored factor for the 3D printing in CHDs. The clinical team attested the low resolution in the reproduction of complex and small anatomical details of FDM printed models, together with the worst tactile feedback due to both the surface roughness and the response to the surgical incision. Although a good resolution of 3D printed models was obtained by SLA technology, the impossibility of a perfect removal of the imprints of external supports negatively affected the surface finish (Figure 3A). Moreover, the simulation of the surgical procedure and the exploration of internal cavities was not always feasible or positively evaluated by the surgical team due to the presence of internal residual supports. On the other hand, the tactile feedback and the response to surgical incision were adequate for all clinicians. Similar behavior was recorded for SLS models, which provided, in addition, the best model quality both in terms of surface finish and accuracy in anatomical detail reproduction. Moreover, the low value of the resulting standard deviation (Figure 3A) denotes an agreement within the clinicians in the evaluation of 3D printing techniques. On the basis of the above reported results, the 3D printed models obtained with SLS technology turned out to be the most suitable for the surgical planning of complex CHDs.

The surgical pre-planning phase of complex CHDs is challenging and time-consuming due to anatomies characterized by uncommon anatomic relations, morphological abnormalities, and often very small structures that can be very dissimilar among the patients (Festa et al., 2023). The inclusion of SLS-based 3D printed models in the pre-planning allows the 3D visualization of the anatomical structures from different perspectives, the manipulation of 1:1 scaled geometries, the exploration of the heart chambers from a surgical point of view, the visualization of the vessels, and chamber spatial relationships. All these aspects have a relevant impact on the decision-making procedure and surgical strategy, bringing an added value in terms of understanding, communication, and time-saving as arisen from clinicians’ response (Figures 3B–D). Regarding the time for the 3D printed model realization, the duration of the segmentation phase is a crucial step that is significantly dependent on the quality of image dataset, the complexity of investigated scenario, and the competences and experience of the multidisciplinary team. The resulting segmentation times in this study were obtained by highly specialized biomedical engineers in the cardiovascular field focused on cardiac structures with more than 5 years of experience. Even if the artificial intelligence applied to image processing is an emerging instrument to obtain an automatic and faster segmentation mask (Garzia et al., 2023), its adoption in the complex CHDs is challenging (Karimi-Bidhendi et al., 2020; Yao et al., 2023) due to the structure relations and abnormalities that can be different for each case. Although the 3D printed models turn out to be a powerful tool to improve the pre-planning procedure for the complex CHDs, their inclusion in the clinical setting is still limited due to the associated costs and time. These aspects could be significantly improved if the facilities required by the 3D printing procedure are present inside the clinical structures without using external service.

## 5 Conclusion

Three-dimensional printing represents an effective instrument for the management of complex CHDs. Among the main diffused technologies, the SLS resulted to be the most suitable for the understanding and planning of surgical procedures, especially for the accuracy of detail reproduction and for the best tactile feedback. The effectiveness of 3D printed models obtained with SLS technology arose from clinicians’ response to a specific satisfaction survey. The presented work demonstrated the improvement provided by 3D printed models in the process of decision-making in the field of complex CHDs.

## Data Availability

The datasets presented in this article are not readily available because the raw data contains sensitive data. Requests to access the datasets should be directed to the corresponding author.
