# Supplementary material for: Three-dimensional printed models as an effective tool for the management of complex congenital heart disease
Source: Front Bioeng Biotechnol. 2024 Aug 2;12:1369514. doi: 10.3389/fbioe.2024.1369514 (PMC11327011; doi:10.3389/fbioe.2024.1369514)
Supplement: Supplementary file 1 [file DataSheet1.PDF]

Supplementary Material

1 SUPPLEMENTARY TABLES AND FIGURES

		FDM	SLA	SLS
Materials		TPU Elasto85, PVA SSU04	Flexible 80A	Flexa Bright
Shore		85A	80A	79A
Layer thickness (mm)		0.150 - 0.250	0.100	0.125
Printing volume (cm^3)		30.0×17.1×20.0	33.5×20.0×30.0	11.0×15.0×25.0
Material cost (€/kg)		104.00, 121.00	242.80	250.00
Equipment cost (€/h)	Printing	0.80	1.25	1.70
	Post-processing	0.10	0.42	0.36

Table S1. Main features of 3D printing technologies adopted in this study.
